# ABC-transporter upregulation mediates resistance to the CDK7 inhibitors THZ1 and ICEC0942

**DOI:** 10.1038/s41388-019-1008-y

**Published:** 2019-09-17

**Authors:** Georgina P. Sava, Hailing Fan, Rosemary A. Fisher, Sabrina Lusvarghi, Sunil Pancholi, Suresh V. Ambudkar, Lesley-Ann Martin, R. Charles Coombes, Lakjaya Buluwela, Simak Ali

**Affiliations:** 1 Division of Cancer, Department of Surgery & Cancer, Imperial College London, Hammersmith Hospital Campus, London, UK; 2 Laboratory of Cell Biology, Center for Cancer Research, National Cancer Institute, Bethesda, MD 20892 USA; 3 The Breast Cancer Now Toby Robins Research Centre, The Institute of Cancer Research, London, UK

**Keywords:** Targeted therapies, Breast cancer

## Abstract

The CDK7 inhibitors (CDK7i) ICEC0942 and THZ1, are promising new cancer therapeutics. Resistance to targeted drugs frequently compromises cancer treatment. We sought to identify mechanisms by which cancer cells may become resistant to CDK7i. Resistant lines were established through continuous drug selection. ABC-transporter copy number, expression and activity were examined using real-time PCR, immunoblotting and flow cytometry. Drug responses were measured using growth assays. ABCB1 was upregulated in ICEC0942-resistant cells and there was cross-resistance to THZ1. THZ1-resistant cells upregulated ABCG2 but remained sensitive to ICEC0942. Drug resistance in both cell lines was reversible upon inhibition of ABC-transporters. CDK7i response was altered in adriamycin- and mitoxantrone-resistant cell lines demonstrating ABC-transporter upregulation. ABCB1 expression correlated with ICEC0942 and THZ1 response, and ABCG2 expression with THZ2 response, in a panel of cancer cell lines. We have identified ABCB1 upregulation as a common mechanism of resistance to ICEC0942 and THZ1, and confirmed that ABCG2 upregulation is a mechanism of resistance to THZ1. The identification of potential mechanisms of CDK7i resistance and differences in susceptibility of ICEC0942 and THZ1 to ABC-transporters, may help guide their future clinical use.

## Introduction

Cyclin dependent kinase (CDK7), along with cyclin H and the accessory protein MAT1, forms the CDK-activating kinase (CAK), which functions to activate CDKs by T-loop phosphorylation [1]. CAK activity directs cell cycle progression via phosphorylation of CDK1, CDK2, CDK4 and CDK6 [2]. CAK is also a component of the general transcription factor TFIIH, which is recruited to transcription start sites, along with the other components of the basal transcriptional machinery, where it phosphorylates RNA polymerase II (Pol II) at serine-5 and serine-7 in its C-terminal domain (CTD) heptapeptide repeats [3]. This Pol II CTD phosphorylation is required for transcription initiation, and therefore, together with its role in regulating the cell cycle, CDK7 is also critical for regulating transcription. CDK9, a component of positive transcription elongation factor (P-TEFb), is likewise activated by CDK7, which in turn results in phosphorylation of the Pol II CTD at serine-2, enabling transcription elongation [4]. In addition, CDK7 also plays a role in activating multiple transcription factors, including the retinoic acid [5], oestrogen [6] and androgen receptors [7], and p53 [8].

Cell cycle deregulation is a common feature of cancer and a number of cell cycle CDK inhibitors have been approved as cancer therapeutics, most prominently CDK4/6 inhibitors, exemplified by palbociclib, for the treatment of advanced oestrogen receptor positive (ER+) breast cancer [9]. By contrast, it was initially thought that the essential nature of transcription would make this process a poor cancer therapeutic target, where transcriptional inhibitors would result in general toxicity and a limited therapeutic window. However, it has now been shown that many tumour types are more heavily dependent than normal tissues on the activity of key transcriptional drivers, for example RUNX1 in acute lymphoblastic leukaemia (ALL) [10] and N-myc in neuroblastoma [10], and also on super-enhancer driven transcription [10–13]. Recently, these findings have become embodied in a wider framework known as “Transcription addiction in cancer” [14], a concept that supports CDK7 as a potentially valuable cancer drug target, as it plays key roles in both the regulation of transcription and the cell cycle. We reported the first selective CDK7 inhibitor, BS-181, as an ATP-competitive CDK7 inhibitor [15], which served as a lead compound for development of the oral clinical candidate ICEC0942 [16], which is currently undergoing Phase I trial for advanced solid malignancies, under the name CT7001. A second, covalent CDK7 inhibitor, THZ1 [10], has been independently developed and a THZ1-derived compound SY-1365 [17] is also undergoing Phase I trial for advanced solid malignancies.

The emergence of resistance to cancer treatment remains a major problem in cancer therapy. Cancer cells can adapt and become resistant to drugs in a multitude of ways, including, but not limited to, mutations that disrupt drug-target engagement, activating mutations in drug targets and downstream pathways, changes in gene expression levels of targets, altered drug metabolism and alterations in cell death pathways [18]. In addition, the upregulation of ATP-dependent efflux pumps with broad substrate specificity can result in multi-drug resistance. The most well-described examples of these are: ATP-binding cassette subfamily B member 1 (ABCB1), also known as multidrug resistance protein 1 or P-glycoprotein; ATP-binding cassette subfamily C member 1 (ABCC1), or multidrug resistance-associated protein 1, and ATP-binding cassette subfamily G member 2 (ABCG2), also known as breast cancer resistance protein [19]. When these ABC-transporter pumps are upregulated, drugs that are substrates are actively effluxed from cancer cells, resulting in decreased intracellular drug accumulation.

Whilst CDK7 inhibitors (CDK7i) represent a promising therapeutic approach, development of resistance in the clinic is possible, therefore identifying potential resistance mechanisms preemptively is of great importance. We have developed models of acquired resistance to both ICEC0942 and THZ1 in the breast cancer cell line MCF7, identifying upregulation of ABC-transporters as mediators of resistance. These findings may be informative for future patient selection strategies and combination treatment regimes.

## Results

### Creation of breast cancer cell lines with resistance to THZ1 and ICEC0942

To investigate mechanisms of acquired resistance to the CDK7i ICEC0942 and THZ1, we generated MCF7-derived drug resistant cell lines through prolonged culturing in the presence of ICEC0942 or THZ1. THZ1 resistance was achieved by initially exposing the cells to 50 nM THZ1, approximately the concentration required for 50% growth inhibition. This was increased gradually up to a concentration of 250 nM, with passaging once a week, until the cells became stably THZ1-resistant after 3 months. A different strategy was used to achieve ICEC0942 resistance, as even cells exposed to drug concentrations well below IC_50_ became growth-arrested after 1–2 weeks, so could not be continually passaged. Cells treated with 800 nM ICEC0942 remained growth arrested until resistance developed after 4 months, eventually giving rise to the ICEC0942-resistant line (MCF7-942^R^).

**Fig. 1:**
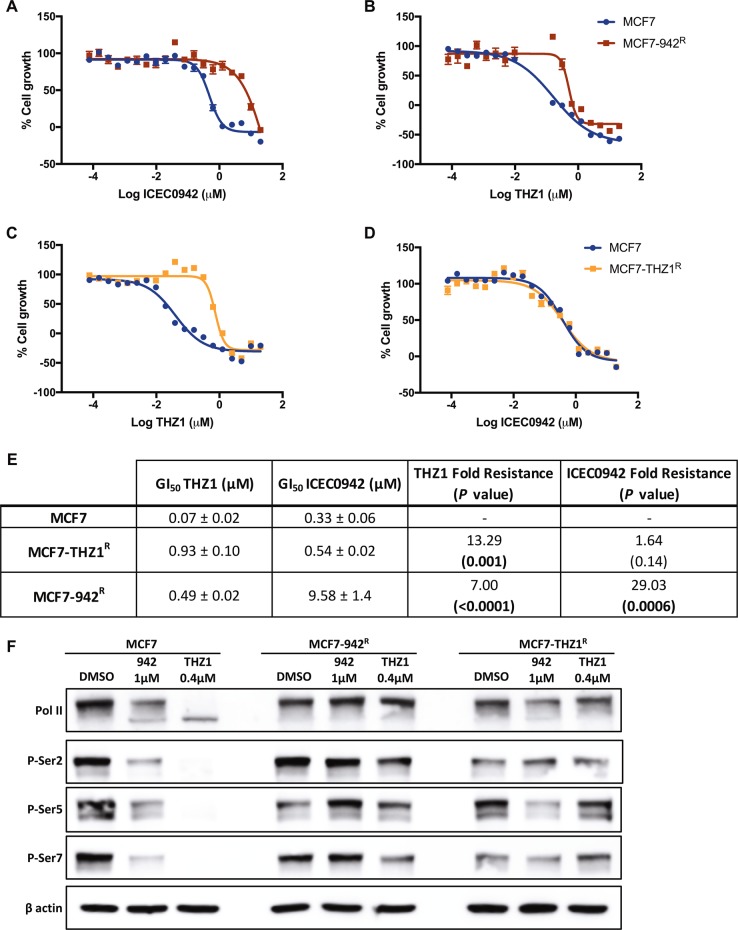
Cross-resistance of ICEC0942- and THZ1- resistant breast cancer cell lines. MCF7, MCF7-942^R^ and MCF7-THZ1^R^ were treated with increasing concentrations of ICEC0942 (**a**, **d**) or THZ1 (**b**, **c**) for 48 h. Mean growth is shown relative to that for vehicle (DMSO)-treated cells. Dose-response curves shown are from single, representative experiments. Average GI_50_ values of the dose response curves (±SEM, *n* = 3 independent experiments) of ICEC0942 and THZ1, and fold-resistance of MCF7-942^R^ and MCF7-THZ1^R^, relative to MCF7, are summarised in **e**. *P* values indicate the statistical significance of the fold resistance. Protein lysates from MCF7, MCF7-942^R^ and MCF7-THZ1^R^, treated with THZ1 or ICEC0942 at indicated concentrations for 24 h, were immunoblotted for Pol II and for phosphorylation of serine-2, serine-5 and serine-7 in the Pol II C-terminal domain (representative of three independent experiments; see Supplementary Fig. S2 for quantification) (**f**)

Compared with MCF7, MCF7-942^R^ showed a 29-fold resistance to ICEC0942 (*P* *=* 0.0006; Fig. 1a, e), and the THZ1-resistant cell line (MCF7-THZ1^R^) showed a 13-fold resistance to THZ1 (*P* *=* 0.001; Fig. 1c, e). MCF7-942^R^ also exhibited significant cross-resistance to THZ1, with a sevenfold increase in GI_50_ (*P* < 0.0001; Fig. 1b, e). Interestingly however, MCF7-THZ1^R^ remained sensitive to ICEC0942 (Fig. 1d, e). We examined Pol II CTD phosphorylation by western blotting after treatment with 1 µM ICEC0942 or 0.4 µM THZ1, concentrations at which maximal differences in growth inhibition between resistant and parental cell lines are observed (Fig. 1a–d). Quantification of three independent experiments showed that ICEC0942 and THZ1 treatment significantly reduced phosphorylation of serine-2, serine-5 and serine-7 in MCF7, whereas in MCF7-942^R^, Pol II CTD phosphorylation was unaffected. However, in MCF7-THZ1^R^, whilst Pol II CTD phosphorylation was unaltered after THZ1 treatment, phosphorylation was reduced after ICEC0942 treatment (Fig. 1f and Supplementary Fig. S1).

THZ1 inhibits CDK7 by covalently targeting a cysteine residue (C312), which lies outside the ATP-binding domain [10], whereas ICEC0942 is an ATP-competitive CDK7i [16]. It has been shown that mutation of C312 to serine (C312S), is sufficient to prevent THZ1 from covalently binding to CDK7 and from inhibiting CDK7 activity [10], therefore it was possible that a mutation of C312 could be responsible for the THZ1-resistance and continued sensitivity to ICEC0942 in MCF7-THZ1^R^. However, DNA sequencing confirmed no sequence change between MCF7 and MCF7-THZ1^R^ at C312 (Supplementary Fig. S2). Subsequently, the full coding sequence of CDK7 was sequenced in MCF7, MCF7-942^R^ and MCF7-THZ1^R^ and no mutations in CDK7 were identified (data not shown).

### THZ1 and ICEC0942 resistance leads to increased expression of ABC-transporters

**Fig. 2:**
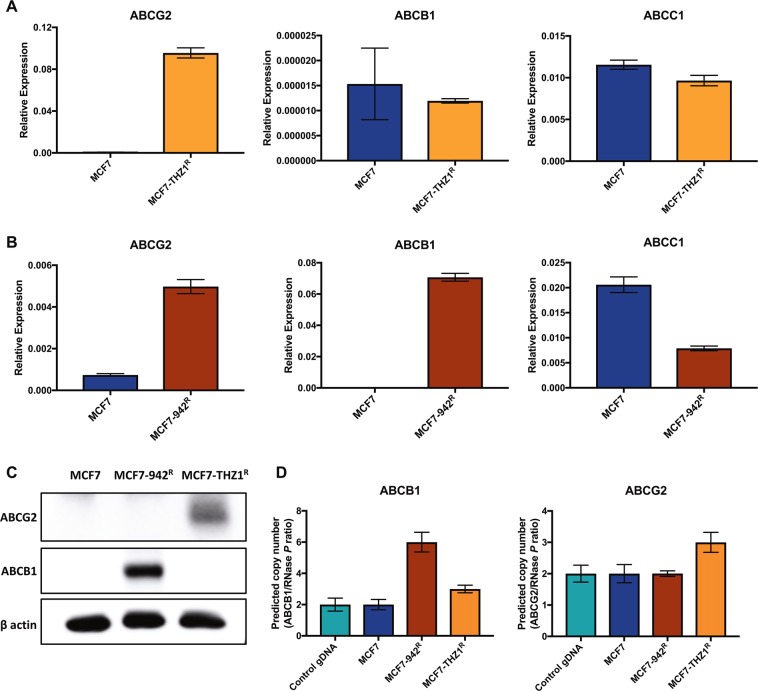
Increased expression of ABC-transporters in ICEC0942- and THZ1- resistant cell lines. mRNA levels of ABCB1, ABCG2 and ABCC1 were compared in MCF7, MCF7-THZ1^R^ (**a**) and MCF7-942^R^ (**b**) by qRT-PCR (error bars = SEM; *n* *=* 3). ABCB1 and ABCG2 protein expression were compared in MCF7, MCF7-942^R^ and MCF7-THZ1^R^ by immunoblotting (**c**). ABCB1 and ABCG2 copy number were compared across the cell lines, and in control genomic DNA, by TaqMan-based copy number analysis (**d**). Predicted copy number, relative to the RNase *P* gene, was calculated using CopyCaller^®^ Software (error bars = SD; *n* *=* 4)

Since upregulation of ABC-transporters is a common mechanism of cancer drug resistance, we used qRT-PCR to examine the mRNA levels of the most frequently observed transporters, ABCB1, ABCC1 and ABCG2, in our CDK7 inhibitor-resistant cell lines. ABCG2 and ABCB1 expression was absent/very low in MCF7 cells, but there was appreciable expression of ABCC1 (Fig. 2a, b). While ABCB1 and ABCC1 expression was unchanged in MCF7-THZ^R^ cells, ABCG2 expression was increased 140-fold, relative to MCF7 cells (Fig. 2a). ABCG2 expression was also around sevenfold higher in In MCF7-942^R^, although its expression remained low. Importantly, there was considerable increase in ABCB1 expression in MCF7-942^R^ (Fig. 2b). Western blotting confirmed the upregulation of ABCB1 in MCF7-942^R^ and of ABCG2 in MCF7-THZ1^R^ (Fig. 2c), while the low-level increase in ABCG2 expression cells was not detectable in MCF7-942^R^ cells.

We next used quantitative gene copy number measurement to determine if the upregulation in ABCB1 and ABCG2 expression was reflected in amplification of the ABCB1 and ABCG2 genes in the resistant cell lines. The gene copy number analysis suggests that there is modest gene amplification in the resistant cell lines, with an estimated six copies of ABCB1 in MCF7-942^R^ and three copies of ABCG2 in MCF7-THZ1^R^, relative to two copies of both genes in MCF7 (Fig. 2d). In MCF7-THZ1^R^ there was also a predicted amplification of ABCB1, however this is not reflected in the mRNA or protein (Fig. 2a, c and d). Similarly, an increase in ABCG2 mRNA expression seen in MCF7-942^R^ is not reflected at the protein level nor at the level of gene copy number increase.

### ABC-transporter mediated efflux is increased in THZ1- and ICEC0942-resistant cell lines

**Fig. 3:**
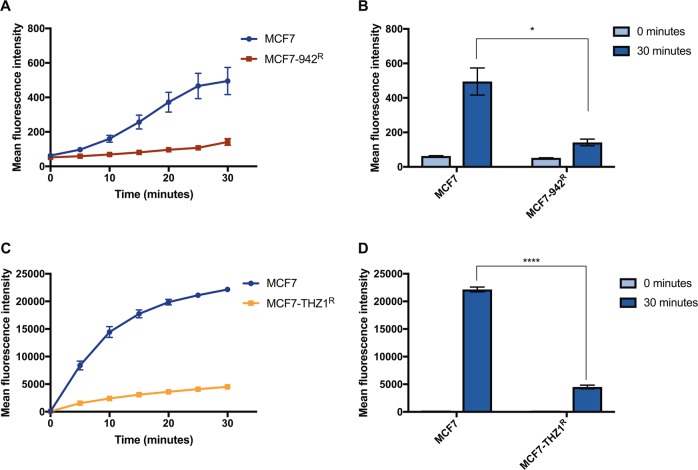
ABC-transporter mediated efflux is altered in ICEC0942-resistant cells. MCF7 and MCF7-942^R^ were incubated with EFFLUXX-ID dye (**a**), and MCF7 and MCF7-THZ1^R^ with Hoechst 33342 (**c**), and the accumulation of intracellular fluorescence was measured by flow cytometry every 5 min for 30 min (error bars = SEM, *n* = 3). The mean fluorescence intensity at time 0 and 30 min is shown for MCF7 vs. MCF7-942^R^ (**b**) and MCF7 vs. MCF7-THZ1^R^ (**d**) (error bars = SEM, *n* = 3). Asterisks represent statistically significant differences (unpaired *t*-test, *P < 0.05, ****P *<* 0.0001)

To assess whether upregulation of ABC-transporters in MCF7-942^R^ and MCF7-THZ1^R^ corresponds with their increased efflux activity, we performed flow cytometry with fluorescent ABC-transporter substrates, assessing intracellular fluorescence as a reflection of pump activity. After 30 min of incubation with the detection reagents, both resistant cell lines exhibited significantly diminished fluorescence in comparison with MCF7, revealing an increase in ABC-transporter activity (Fig. 3a–d).

### CDK7 inhibitor resistance can be reversed by ABC-efflux pump inhibitors

**Fig. 4:**
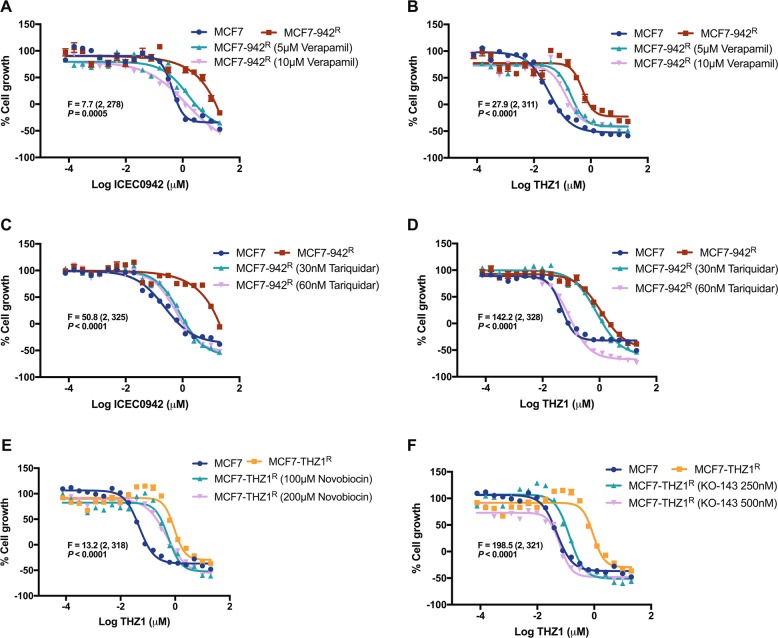
ICEC0942 and THZ1 resistance can be reversed by ABC-transport inhibitors. MCF7, and MCF7-942^R^ were treated with increasing concentrations of ICEC0942 or THZ1 for 48 h with addition of the ABCB1 inhibitors, verapamil or tariquidar (at indicated concentrations), or with vehicle (DMSO) (**a**–**d**). MCF7, and MCF7-THZ1^R^ were treated with increasing concentrations of THZ1 for 48 h with addition of the ABCG2 inhibitors, novobiocin (**e**) or KO-143 (**f**) (at indicated concentrations), or with vehicle (DMSO). The sum-of-squares *F* test was used to assess whether the addition of ABCB1/ABCG2 inhibitors significantly affected the GI_50_ of ICEC0942 and/or THZ1, in MCF7-942^R^ and MCF7-THZ1^R^. The corresponding *F* (degrees of freedom in brackets) and *P* values are presented (**a**–**f**). Dose-response curves shown are from single experiments (error bars = SEM, *n* = 6). For clarity, the vehicle control growth curves for MCF7 and MCF7-THZ1^R^ have been presented in both **e**, **f**

To explore whether the increase in ABC-transporter activity in the CDK7 inhibitor-resistant cell lines is responsible for their drug resistance, we utilised specific inhibitors of ABCB1 and ABCG2. ICEC0942-resistant cells were treated with the ABCB1-specific inhibitors verapamil [20] and tariquidar [21], alongside increasing concentrations of ICEC042 or THZ1. Both ABCB1 inhibitors increased the sensitivity of MCF7-942^R^ cells to ICEC0942 and THZ1 in a dose dependent manner (Fig. 4a–d). THZ1-resistant cells were treated with the ABCG2-specific inhibitors novobiocin [22] and KO-143 [23], in combination with increasing concentrations of THZ1. Likewise, both ABCG2 inhibitors dose-dependently reduced the GI_50_ of THZ1 in MCF7-THZ1^R^ (Fig. 4e, f), albeit the increase in sensitivity to THZ1 was more pronounced for KO-143, than for novobiocin, which is unsurprising considering KO-143 has previously been shown to be a more potent modulator of ABCG2 [24]. To further confirm the role of ABC-transporters in CDK7i resistance, we carried out siRNA knockdown experiments. The siRNA-mediated reduction of ABCB1 in MCF7-942^R^ and of ABCG2 in MCF7-THZ1^R^ was confirmed by qRT-PCR and western blot (Supplementary Fig. S3A–D). In MCF7-942^R^, knockdown of ABCB1 resensitised cells to both ICEC0942 and THZ1 (Supplementary Fig. S3F, G). Similarly, in MCF7-THZ1^R^, knockdown of ABCG2 resensitised cells to THZ1 (Supplementary Fig. S3H).

To confirm if ABCB1 upregulation is common, we cultured an additional independent MCF7 cell stock in ICEC0942, to generate resistance. ABCB1 was similarly also upregulated in resistance to ICEC0942 (Supplementary Fig. S4A). ICR-MCF7-942^R^ had reduced sensitivity to both ICEC0942 and THZ1, in comparison with its parental cell line, ICR-MCF7 (Supplementary Fig. S4B–E). Like MCF7-942^R^, treatment of ICR-MCF7-942^R^ with verapamil or tariquidar, alongside increasing concentrations of ICEC042 or THZ1, could resensitise the cells to the CDK7i (Supplementary Fig. S4B–E).

To ascertain whether ABC-transporter upregulation alone, is sufficient for resistance to CDK7i, we utilised HEK293 cells stably overexpressing either ABCB1 (HEK293^ABCB1^), ABCG2 (HEK293^ABCG2^) or empty vector (HEK293) (Supplementary Fig. S5A). Overexpression of ABCB1 caused resistance to both THZ1 and ICEC0942. However, whilst overexpression of ABCG2 resulted in resistance to THZ1, HEK293^ABCG2^ remained sensitive to ICEC0942 (Supplementary Fig. S5B, C).

Docking studies were performed to investigate the possible interactions of ICEC0942 and THZ1 with the drug-binding pockets of ABCB1 and ABCG2. Whilst both compounds are likely to interact with ABCB1 and ABCG2, THZ1 appears to have a stronger overall affinity for both transporters (Supplementary Fig. S6).

### ABCB1 and ABCG2 are correlated with CDK7 inhibitor response in a cancer cell line panel

**Fig. 5:**
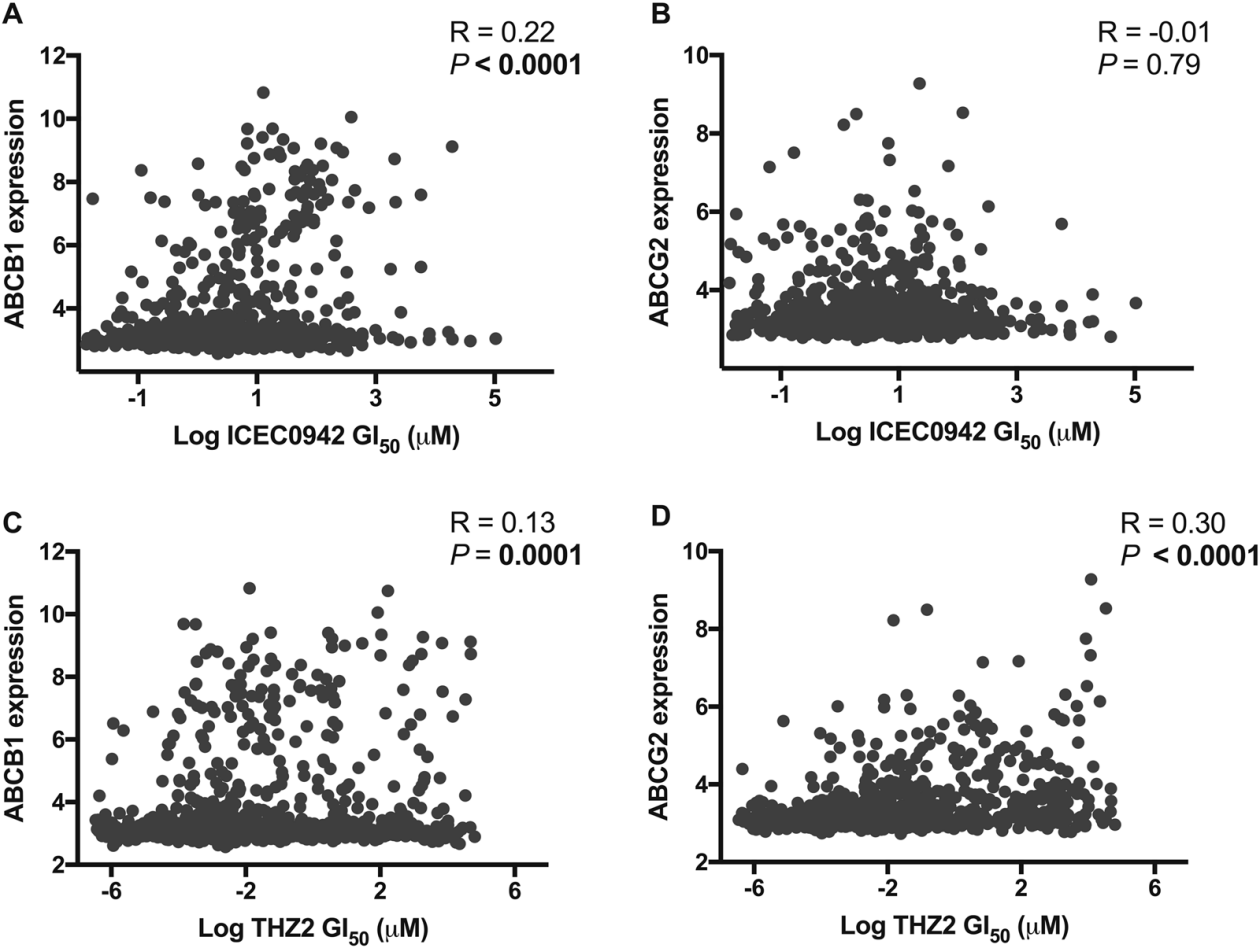
Expression of ABC-transporters is correlated with CDK7 inhibitor response in a cancer cell line panel. A panel of 854 cancer cell lines was screened with ICEC0942 and THZ2 and the GI_50_ values for each cell line plotted against their mRNA expression levels of ABCB1 (**a**, **c**) and ABCG2 (**b**, **d**). *R* = Pearson correlation coefficient

To establish wider evidence for a relationship between ABCB1 expression level and ICEC0942 response, we examined the correlation between mRNA levels of ABCB1 and ICEC0942 GI_50_ in a panel of 854 cancer cell lines representing a variety of cancer types [25]. A modest but significant, positive correlation between ABCB1 expression and ICEC0942 GI_50_ was observed (*P* *<* 0.0001; Fig. 5a). In contrast, we saw no correlation between ICEC0942 GI_50_ and ABCG2 expression levels (*P* = 0.79; Fig. 5b).

Although screening data was not available for THZ1, THZ2, a THZ1-derived analogue with similar CDK7 selectivity [26], has been screened using this cancer cell line panel. An examination of the THZ2 cell line sensitivity data revealed moderate, but significant positive correlations between expression levels of both ABCB1 (*P* = 0.0001) and ABCG2 (*P* *<* 0.0001) and THZ2 response (Fig. 5c, d).

### Chemotherapy-resistant cells are cross-resistant to CDK7 inhibitors

**Fig. 6:**
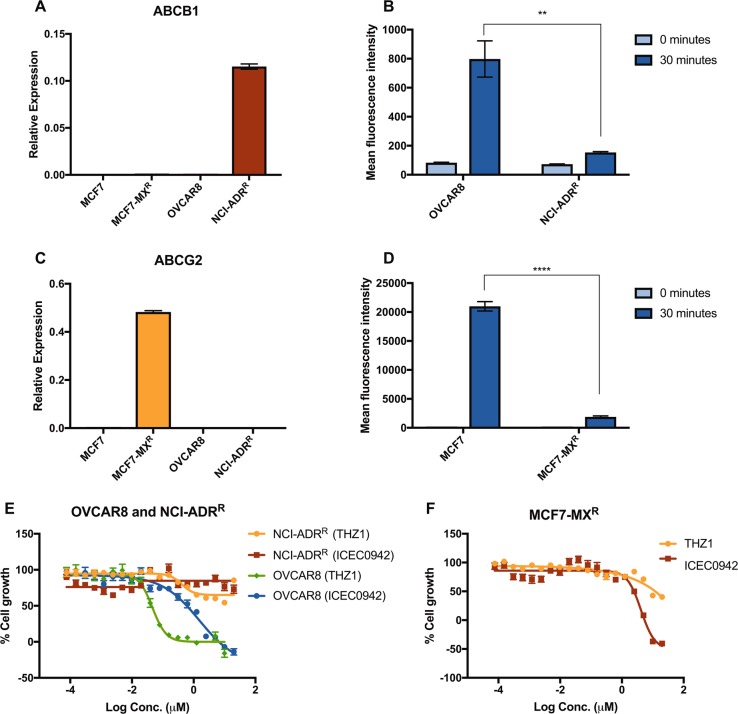
Chemotherapy-resistant cell lines are cross-resistant to CDK7 inhibitors. mRNA levels of ABCB1 (**a**) and ABCG2 (**c**) were examined in OVCAR8, NCI-ADR^R^, MCF7 and MCF7-MX^R^ by qRT-PCR (error bars = SEM, *n* = 3). OVCAR8 and NCI-ADR^R^ were incubated with EFFLUXX-ID dye and MCF7 and MCF7-MX^R^ with Hoechst 33342, and the accumulation of intracellular fluorescence was measured by flow cytometry every 5 min for 30 min. The mean fluorescence intensity at time 0 and 30 min is shown for OVCAR8 vs. NCI-ADR^R^ (**b**) and MCF7 vs. MCF7-MX^R^ (**d**) (error bars = SEM, *n* = 3). NCI-ADR^R^ (**e**) and MCF7-MX^R^ (**f**) were treated with increasing concentrations of THZ1 or ICEC0942 for 48 h. Dose-response curves shown are from single experiments (error bars = SEM, *n* = 6). Mean growth is shown relative to that for vehicle (DMSO)-treated cells. Asterisks represent statistically significant differences (unpaired *t*-test, **P < 0.01, ****P *<* 0.0001)

As upregulation of ABC-transporters represents a mechanism of resistance common to a wide range of cancer drugs, we wondered whether cell lines with pre-established resistance to chemotherapeutics would also be cross-resistant to CDK7i. Here we utilised an adriamycin-resistant cell line from the NCI-60 panel, derived from the ovarian cancer line OVCAR8 (NCI-ADR^R^) [27] and a mitoxantrone-resistant MCF7-derived cell line (MCF7-MX^R^) [28, 29]. qRT-PCR confirmed that ABCB1 is highly expressed in NCI-ADR^R^ (Fig. 6a) and flow cytometry with a fluorescent ABCB1 substrate (as described above) verified efflux pump activity (Fig. 6b). NCI-ADR^R^ is completely non-responsive to both THZ1 and ICEC0942, even at the highest doses tested (20 µM) (Fig. 6e), whereas OVCAR8 cells are sensitive to ICEC0942 and THZ1, with a GI_50_ of 1.5 µM ± 0.09 and 0.05 µM ± 0.02, respectively (Fig. 6e).

MCF7-MX^R^ was confirmed to have high expression and pump activity of ABCG2, by qRT-PCR (Fig. 6c) and flow cytometry with fluorescent detection reagents (Fig. 6d). This cell line displayed resistance to THZ1, with the highest dose (20 µM) achieving only around a 50% reduction in cell growth (Fig. 6f). However, ICEC0942 induced growth inhibition and cell death in MCF7-MX^R^ at doses above 625 nM (Fig. 6f).

## Discussion

Our findings have identified upregulation of ABC-transporters as a potential mechanism of acquired resistance to CDK7i that are in clinical evaluation. Specifically, upregulation of ABCB1 can mediate resistance to both the ATP-competitive inhibitor ICEC0942, and the covalent inhibitor THZ1, whereas ABCG2 upregulation results in THZ1-resistance whilst ICEC0942-sensitivity is maintained.

MCF7 cells with acquired resistance to ICEC0942 were developed, which were cross-resistant to THZ1, showed amplification of the ABCB1 gene and correspondingly, increased ABCB1 efflux activity. A competitive inhibitor of ABCB1, verapamil [20], and a non-competitive inhibitor, tariquidar [30], were able to resensitise the cells to both CDK7i. This provides evidence for the biological relevance of ABCB1 activity in the drug resistance of this cell line. In further support of this, siRNA knockdown of ABCB1 was also able to reverse the resistance to CDK7i, and ABCB1 overexpression was able to induce resistance to CDK7i. Prolonged treatment of MCF7 cells with THZ1 resulted in a THZ1-resistant cell line with gene amplification of ABCG2 and a corresponding upregulation of ABCG2. Importantly, despite an over thirteen-fold resistance to THZ1, these cells remained sensitive to ICEC0942. Again, sensitivity to THZ1 in this cell line was restored with inhibitors of ABCG2, and also with siRNA knockdown of ABCG2, whereas ABCG2 overexpression could generate resistance to THZ1. This provides support for a mechanistic role of ABCG2 in mediating THZ1-resistance.

In a drug screen on a large cancer cell line panel representing multiple tumour types [25], a significant correlation between the expression of ABCB1 and response to ICEC0942 was observed across the panel. No screening data were available for THZ1 in this cell line panel, hence we made use of cell line response data for THZ2, an analog of THZ1, which is also selective for CDK7 [26]. ABCB1 expression also correlated significantly with THZ2 response, as did the expression of ABCG2, whereas no correlation between ABCG2 expression and ICEC0942 sensitivity was detected. Whilst providing support for the role of ABC-transporters in mediating CDK7 inhibitor response, this analysis was based on intrinsic gene expression, whereas the majority of this study pertains to ABC-transporter upregulation in the acquired resistance setting. In addition, a cell line with previously acquired adriamycin resistance and high ABCB1 expression was completely resistant to both CDK7i, whereas a mitoxantrone-resistant cell line with high ABCG2 expression, although non-responsive to THZ1, retained some ICEC0942-sensitivity. Taken together these results suggest that ICEC0942 and THZ1 are both substrates for ABCB1 transport, and ABCB1 upregulation can mediate resistance to both drugs. On the other hand, while THZ1 represents a substrate for ABCG2 transport, ICEC0942 is less readily effluxed by ABCG2, and ABCG2 upregulation can mediate resistance to THZ1. Docking studies suggest that ICEC0942 and THZ1 can both interact with ABCB1 and ABCG2, THZ1 with a stronger affinity than ICEC0942. These studies, however, did not provide insight into structural differences that could explain the difference in affinity of ABCG2 for THZ1 over ICEC0942. Hydrophobic regions have been identified as a feature common to all ABCG2 substrates [31], so it is perhaps of note that THZ1 has a higher cLogP value than ICEC0942 (6.33 and 2.85, respectively) [32], perhaps offering some explanation for the differing potential of the compounds for ABCG2-mediated transport. Nevertheless, the differential sensitivity to ABCG2 may allow broader use of ICEC0942 than THZ1 and/or aid sequencing of the two drugs.

Despite great advances in the development of targeted anticancer agents, drug resistance remains a major problem that ultimately limits the effectiveness of almost all known cancer therapeutics [33]. The recently developed CDK7i, ICEC0942 and THZ1, have shown great promise for the treatment of multiple tumour types, including breast cancer. CDK7 inhibition inhibits the growth of ESR1-mutant breast cancer [34, 35] and can overcome chemotherapy resistance in breast cancer [36]. Combinations of CDK7i with other cancer therapeutics have also been explored, for example, BRD4 and CDK7i have been shown to work synergistically in neuroblastoma [37] and a combination of PARP and CDK7i show synergism for the treatment of Ewing Sarcoma [38]. Both ICEC0942 and SY-1365, a drug related to THZ1, are currently undergoing phase I clinical trials for advanced malignancies; however, it is likely that even if they are successfully approved as cancer therapies, drug resistance will be an issue, therefore identifying potential resistance mechanisms is crucial if this is to be overcome.

Gao et al., previously identified ABCB1 and ABCG2 upregulation as mechanisms of resistance to THZ1 in neuroblastoma and lung cancer cells [39]. Our study confirms these mechanisms and extends these findings to breast cancer. This is the first study designed to investigate mechanisms of resistance to ICEC0942, although both ABCB1 and ABCG2 have been implicated in resistance to the pan-CDK inhibitor BS-194, a chemically-related precursor to ICEC0942 [40]. Our present study suggests that the structural differences between BS-194 and ICEC0942 have resulted in a reduced affinity of ABCG2 for ICEC0942 compared with BS-194.

Despite the multiple lines of evidence that we present, demonstrating the role of ABC-transporters in the CDK7 inhibitor-resistance of our cell line models, it is conceivable that other unidentified mechanisms of resistance may also be involved, for example, gene expression changes or mutations in genes other than CDK7. However, the ability of ABC-transport inhibition by small molecules and siRNA knockdown to reverse their drug-resistant phenotypes, strongly suggests that even if other mechanisms pertaining to CDK7 inhibitor resistance exist in these cells, overactivity of ABC-transporters provides the predominant mode of resistance.

The ability of cancer cells to acquire resistance to multiple structurally and mechanistically unrelated drugs severely limits treatment success in patients [19]. The involvement of ABC-transporters in drug resistance was initially identified in cell lines [41–43], but they have since been implicated in some clinical settings. Overexpression of ABCB1 has been shown to be associated with drug resistance in a number of different tumour types [44], including ceritinib resistance in non-small cell lung cancer [45], and with poor clinical outcomes, such as response to chemotherapy in breast cancer [46]. In addition, gene rearrangements that fuse the ABCB1 gene with an aberrant active promoter that drives its expression, have been observed in ovarian cancer [47, 48], lymphoma [49] and breast cancer [48]. Correlations between ABCG2 expression and decreased survival [50, 51] and response to therapy have been identified, including dasatinib response in chronic myeloid leukaemia [52].

Despite large efforts, clinical trials of ABC-transport inhibitors in combination with antineoplastic drugs have mostly been unsuccessful, due to issues with toxicity, potency and trial design [19]. However, should future developments be made in this area, our findings implicate CDK7i as potential candidates for combination treatments with such ABC-transport inhibitors. Our results suggest that CDK7i may be of most benefit if used before patients have received multiple lines of chemotherapy, as tumours with acquired resistance to other therapeutics may have upregulated ABC-transporters, and so may also be cross-resistant to CDK7i. In this context, tumours with highly upregulated ABCB1 expression may be unlikely to respond to either THZ1 or ICEC0942, whereas tumours with high ABCG2 expression may derive benefit from ICEC0942 treatment.

We acknowledge that this study relies heavily on a single breast cancer cell line model, MCF7. An extension of this work to other tumour types and in vivo studies would be required to further investigate the generality of ABC-transporters in CDK7 inhibitor resistance. It is likely that in the clinical setting, alternative mechanisms of resistance that have not yet been identified will be important, for example genomic changes that activate pathways compensatory for CDK7 inhibition, may play a role. Further studies will be required to identify these and grasp a full understanding of the molecular mechanisms that can give rise to CDK7 inhibitor resistance.

In conclusion, we have identified upregulation of ABC-transporters as a mechanism of resistance to two independent, selective CDK7i. Upregulation of ABCB1 results in resistance to both ICEC0942 and THZ1, whereas ABCG2 upregulation can also mediate THZ1 resistance. These findings have potential implications for patient selection, combination treatment strategies and for CDK7 inhibitor selection, suggesting, for example that it may be beneficial for CDK7i to be used prior to chemotherapies associated with ABC-transporter upregulation.

## Materials and methods

### Cell lines

MCF7 cells were obtained from the ATCC (www.lgcstandards-atcc.org) as frozen stocks. The NCI Adriamycin-resistant cell line (NCI-ADR^R^) was provided by Dr. Ernesto Yagüe, and OVCAR8 by Dr. Paula Cunnea, both from Imperial College London (London, UK). The MCF7 mitoxantrone-resistant cell line (MCF7-MX^R^) was provided by Dr. E Schneider from the University of Maryland (Baltimore, MD, USA). HEK293 cells, stably transfected with either empty pcDNA 3.1 vector (HEK293), pcDNA3.1 containing ABCB1 (HEK293^ABCB1^) or ABCG2 (HEK293^ABCG2^) have been described [53]. All cell lines were maintained in DMEM supplemented with 10% FCS, apart from OVCAR8, which was maintained in RPMI supplemented with 10% FCS, HEK293 lines, which were maintained in MEM supplemented with 10% FCS and 2 mg/ml geneticin, and ICR-MCF7/ICR-MCF7-942^R^, which were maintained in phenol-red free RPMI, supplemented with 10% FCS and 1 nM estradiol. Cell lines were cultured up to a maximum of 20 passages beyond initial thawing. Cell cultures were routinely tested for Mycoplasma infection by assay of culture supernatants, and found to be negative, using the MycoAlert Mycoplasma Detection Kit (Lonza, UK).

### Establishment of drug resistant cell lines

The MCF7 ICEC0942-resistant cell line (MCF7-942^R^) was established by growing MCF7 cells in the continued presence of ICEC0942 at 800 nM for 4 months, splitting when cells neared confluence. The MCF7 THZ1-resistant cell line (MCF7-THZ1^R^) was established by initially growing MCF7 with THZ1 at 50 nM. Cells were split when they neared confluence and the THZ1 concentration then raised by 25 nM increments, up to a maximum of 250 nM, over a 3-month period. The independent MCF7 ICEC0942-resistant cell line (ICR-MCF7-942^R^) was established by growing MCF7 cells in the continued presence of ICEC0942 at 400 nM for 3 months, splitting when cells neared confluence. After drug resistance was established, the cell lines were maintained in culture with 800 nM ICEC0942 (MCF7-942^R^), 400 nM ICEC0942 (ICR-MCF7-942^R^), or 250 nM THZ1 (MCF7-THZ1^R^), with medium and drug changes every 3–4 days.

### Chemicals

ICEC0942 synthesis has been described [54] and was provided by Carrick Therapeutics. THZ1 (A8882) was purchased from ApexBio Technology (Boston, MA, USA). Verapamil (CAY14288), Tariquidar (CAY24180) and KO-143 (CAY15215) were purchased from Cambridge Bioscience (Cambridge, UK). Novobiocin (74675) was purchased from Sigma-Aldrich (Dorset, UK).

### Drug sensitivity assays

The sulphorhodamine B (SRB) assay was used to assess drug sensitivity, as previously described [55]. Drug concentrations that inhibited 50% of cell growth (GI_50_) were calculated from log dose-response curves in GraphPad prism v7.0. All dose-response curves are presented with error bars for standard errors of the mean (SEM) of six technical replicates. A minimum of three independent experiments were conducted and the average fold resistance of the drug resistant cell lines, MCF7-942^R^ and MCF7-THZ1^R^, relative to the parental cell line, MCF7, was determined thus; fold resistance = GI_50_ resistant cell line/GI_50_ parental cell line [56]. For ABCB1 and ABCG2 inhibitor studies, the same procedure was followed, except inhibitors were simultaneously added to cells with THZ1 or ICEC0942. SRB assays with MCF7 were used to identify non-toxic ABCB1/ABCG2 inhibitor concentrations at 48 h, for use in studies (data not shown).

### Immunoblotting

Whole cell lysates were prepared in RIPA buffer (Sigma-Aldrich), supplemented with protease and phosphatase inhibitors (Roche, UK), and immunoblotting was carried out as previously described [16]. Antibodies used were; Pol II (ab26721), Pol II-phosphoserine-2 (ab5095), Pol II-phosphoserine-5 (ab5131), Pol II-phosphoserine-7 (ab126537) and β actin (ab6276), purchased from Abcam (Cambridge, UK), and ABCB1 (D3H1Q) and ABCG2 (4477), purchased from Cell Signaling Technology (Danvers, MA, USA). Band density was quantified using ImageJ software.

### Flow cytometry

The EFLUXX-ID Green Multidrug Resistance Assay Kit (Enzo Life Sciences, Exeter, UK) was used to assess the pump efflux activity of ABCB1 in MCF7, MCF7-942^R^ and NCI-ADR^R^. Cells were trypsinised and resuspended in phenol red-free DMEM at a concentration of 1 × 10^6^ cells per ml. Twenty microliters of the EFFLUXX-ID Green Detection Reagent (in DMSO) was diluted in 1 ml DMEM (phenol red-free). Cells (2 × 10^6^) were incubated with 50 µl of the diluted EFFLUXX-ID Green Detection Reagent and the fluorescence intensity of each sample was measured for 10,000 events every 5 min for 30 min, on a BD LSR II Flow Cytometer (BD Biosciences, San Jose, CA, USA). The same procedure was used to assess the pump efflux activity of ABCG2 in, MCF7, MCF7-THZ1^R^ and MCF7-MX^R^, except Hoechst 33342 [57] was used instead of EFFLUXX-ID, at a final concentration of 5 µg/ml. This was repeated for three replicates. Prior to analysis unstained cells were used for gating. To analyse, the mean fluorescence intensity of each sample was plotted against time, with error bars for SEM.

### siRNA knockdown

Knockdown of ABCG2 in MCF7-THZ1^R^ cells was carried out by reverse transfection, using Lipofectamine RNAiMAX transfection reagent (Thermo Scientific), with ABCG2-specific siRNA (Dharmacon SMARTpool ON-TARGETplus; GE Healthcare Life Sciences, Little Chalfont, UK) or non-targeting siRNA, (Dharmacon ON-TARGETplus Nontargeting Pool) in either six well plates (for RNA and protein expression; 3 × 10^5^ cells per well), or 96 well plates (for drug sensitivity assays; 5000 cells per well), at a final concentration of 25 nM. ABCB1 knockdown in MCF7-942^R^ cells was carried out in the same way but with ABCB1-specific siRNA (Dharmacon SMARTpool ON-TARGETplus) or non-targeting siRNA (Dharmacon ON-TARGETplus Nontargeting Pool). RNA preparation, qRT-PCR, protein preparation, immunoblotting and drug sensitivity assays were then carried out as described above. Primers used in sequencing and qRT-PCR are listed in Supplementary Table S1.

### Statistical analyses

Statistical analyses were carried out in Graphpad prism v7.0. Pairwise comparisons were performed using the Student *t-*test. The Pearson correlation coefficient was used for gene expression analyses. To assess whether ABC-transport inhibitors significantly alter the GI_50_ of CDK7i, in CDK7 inhibitor-resistant cell lines, the extra sum-of-squares *F* test was used.

## Supplementary information

Supplementary Methods.

Supplementary Figure Legends.

Supplementary Figures.
