# Supplementary material for: ABC-transporter upregulation mediates resistance to the CDK7 inhibitors THZ1 and ICEC0942
Source: Oncogene. 2019 Sep 17;39(3):651–63. doi: 10.1038/s41388-019-1008-y (PMC6962093; doi:10.1038/s41388-019-1008-y)
Supplement: Supplementary file 1 — Supplementary Methods. [file 41388_2019_1008_MOESM1_ESM.docx]

## SUPPLEMENTARY METHODS

### Gene expression analyses

854 cancer cell lines from the GDSC1000 panel^5^ were screened with increasing concentrations of ICEC0942, up to a maximum of 10 µM, and the GI_50_ per cell line was estimated (methods described in detail at <https://www.cancerrxgene.org/>). For the same cell line panel, publicly available Affymetrix Human Genome U219 gene expression array data and THZ2 GI_50_ data, was obtained from https://www.cancerrxgene.org/. The relationship between the log GI_50_ of ICEC0942 and THZ2, and RMA normalised mRNA levels of ABCB1 and ABCG2 across the panel of cell lines, was assessed.

### *In silico* modelling

The inward-open structure of human ABCB1 (PDBID:6QEX)^1^ and ABCG2 homodimer (PDBID:5NJ3)^3^, were used for docking of ICEC0492 and THZ1 with AutoDock Vina^4^. Transporters and ligands were prepared using MGLtools software package^2^ (Scripps Research Institute). The side chains of the following 36 residues, all located in the drug-binding pocket in the transmembrane region of ABCB1 were set as flexible: L65, M68, M69, F72, Q195, W232, F303, I306, Y307, Y310, F314, F336, L339, I340, F343, Q347, N721, Q725, F728, F732, F759, F770, F938, F942, Q946, M949, Y953, F957, L975, F978, V982, F983, M986, Q990, F993, F994. The receptor grid was centred at x = 19, y = 53 and Z =3 and a box with inner box dimensions 40 Å x 40 Å x 44 Å was used to search of all the possible binding poses within the transmembrane region of the protein. In the case of ABCG2, the following residues were set as flexible: N393, A397, N398, V401, L405, I409, T413, N424, F431, F432, T435, N436, F439, S440, V442, S443, Y538, L539, T542, I543, V546, F547, M549, I550, L554, L555. The receptor grid was centred at x = 125, y = 125 and Z =130 and a box with inner box dimensions 34 Å x 30 Å x 50 Å. The exhaustiveness level was set at 100 for both proteins to ensure that the global minimum of the scoring function would be found, considering the large box size and the number of flexible residues.

### Fluorescent microsatellite genotyping of cell lines

All cell lines were authenticated by short tandem repeat profiling using an AmpFlSTR Identifiler Plus kit (Applied Biosystems, Warrington, UK). Briefly, DNA (5 ng) was amplified with a panel of primers for 15 short tandem repeat (STR) loci on 13 autosomes and the amelogenin locus, PCR products were resolved by capillary electrophoresis using an ABI 3100 Genetic Analyser and genotypes determined using GeneMapper version 5.0 software (Applied Biosystems).

### DNA sequencing

Genomic DNA was extracted from MCF7 and MCF7‑THZ1^R^ using the PureLink Genomic DNA Mini Kit (Thermo Scientific). A 523 bp PCR product spanning exon 11 of CDK7, which encodes for cysteine 312, was generated and sequenced using the primers listed in Supplementary Table 1. cDNA, reverse transcribed from RNA extracted from MCF7, MCF7‑942^R^ and MCF7‑THZ1^R^ (as described above), was used as template for PCRs with primers targeted to the 3’ and 5’ UTRs of CDK7 (see Supplementary Table 1 for primer sequences). This generated a 1348 bp PCR product, encompassing the entire coding region of CDK7, which was then Sanger sequenced using the primers listed in Supplementary Table 1.

### RNA preparation and quantitative RT‑PCR

Total RNA was extracted from 5 x 10^6^ million cells using the RNeasy Plus Mini Kit (Qiagen, Manchester, UK) and converted to cDNA using random hexamer priming with RevertAid reverse transcriptase (RT) (Thermo Scientific). Quantitative RT‑PCR (qRT‑PCR) was carried out with three technical replicates for each sample using SYBR green DNA dye (Thermo Scientific) and primers listed in Supplementary Table 1. GAPDH primers were purchased from Primerdesign (Hampshire, UK). Results are presented as the mean fold difference to the control (GAPDH), with error bars for SEM.

### Copy number analysis

ABCB1 and ABCG2 copy number were estimated using a quantitative TaqMan gene copy number assay (TaqMan assay IDs Hs04962504_cn and Hs00856157_cn, respectively) (Life Technologies, Paisley, UK). Gene copy numbers for four technical replicates were compared to the control gene RNase P (TaqMan Copy Number Reference Assay) by the comparative Ct method and relative quantitation values obtained using CopyCaller Software (Applied Biosystems, Waltham, MA, USA). As an additional control ABCB1, ABCG2 and RNase P copy number were assessed in TaqMan Control Genomic DNA (Life Technologies).

## REFERENCES

1 Alam A, Kowal J, Broude E, Roninson I, Locher KP. Structural insight into substrate and inhibitor discrimination by human P-glycoprotein. Science (New York, NY) 2019; 363: 753-756.

2 Sanner MF, Olson AJ, Spehner J-C. Reduced surface: An efficient way to compute molecular surfaces. Biopolymers 1996; 38: 305-320.

3 Taylor NMI, Manolaridis I, Jackson SM, Kowal J, Stahlberg H, Locher KP. Structure of the human multidrug transporter ABCG2. Nature 2017; 546: 504.

4 Trott O, Olson AJ. AutoDock Vina: improving the speed and accuracy of docking with a new scoring function, efficient optimization, and multithreading. Journal of computational chemistry 2010; 31: 455-461.

5 Yang W, Soares J, Greninger P, Edelman EJ, Lightfoot H, Forbes S *et al*. Genomics of Drug Sensitivity in Cancer (GDSC): a resource for therapeutic biomarker discovery in cancer cells. Nucleic Acids Research 2013; 41: D955-D961.
