# Supplementary material for: ABC-transporter upregulation mediates resistance to the CDK7 inhibitors THZ1 and ICEC0942
Source: Oncogene. 2019 Sep 17;39(3):651–63. doi: 10.1038/s41388-019-1008-y (PMC6962093; doi:10.1038/s41388-019-1008-y)
Supplement: Supplementary file 2 — Supplementary Figure Legends. [file 41388_2019_1008_MOESM2_ESM.docx]

**Supplementary Figure Legends**

### Supplementary Figure S1. Treatment with ICEC0942 reduces Pol II CTD phosphorylation in MCF7 and MCF7-THZ1R, but not MCF7-942^R^.

MCF7, MCF7-942^R^ and MCF7-THZ1^R^, treated with THZ1 or ICEC0942 at indicated concentrations for 24 hours, were immunoblotted for Pol II and for phosphorylation of serine­­­­­­-2, serine-5 and serine-7 in the Pol II C‑terminal domain. Three independent repeats were carried out and bands were densitometrically quantified using ImageJ software. The mean density, relative to vehicle control, and normalised to β actin, is shown (error bars = SEM, *n* = 3). Asterisks represent statistically significant differences (unpaired *t*-test, * = *P* < 0.05).

### Supplementary Figure S2. CDK7 sequence encompassing C312 in MCF7 and MCF7-THZ1^R^

The genomic sequence of MCF7 and MCF7‑THZ1^R^, aligned with the CDK7 amino acid sequence, for the region surrounding cysteine 312 (C312) (denoted by black box).

### Supplementary Figure S3. Knockdown of ABC‑transporters can reverse ICEC942 and THZ1 resistance.

qRT‑PCR and Western blot confirmed the reduction of ABCB1 expression in MCF7‑942^R^ (A and C) and of ABCG2 in MCF7‑THZ1^R^ (B and D), 72 hours post‑transfection with targeting or non-targeting control siRNA (error bars = SEM; *n* = 3). MCF7‑942^R^ was treated with increasing concentrations of ICEC0942 (E) or THZ1 (F) for 48 hours, after transfection with either non-targeting control siRNA or siRNA targeting ABCB1. MCF7 treated with increasing concentrations of ICEC0942 or THZ1, with no siRNA transfection, is shown for comparison. MCF7‑THZ1^R^ was treated with increasing concentrations of THZ1 (G) for 48 hours, after transfection with either non-targeting control siRNA or siRNA targeting ABCG2. MCF7 treated with increasing concentrations of THZ1, with no siRNA transfection, is shown for comparison. Dose-response curves shown are from single experiments (error bars = SEM, *n* = 6). Mean growth is shown relative to that for vehicle (DMSO)‑treated cells.

### Supplementary Figure S4. ABCB1 upregulation causes CDK7 inhibitor resistance in an independent ICEC0942-resistant cell line.

Western blot confirmed the upregulation of ABCB1 in ICR-MCF7-942^R^ (A). ICR-MCF7 and ICR-MCF7‑942^R^ were treated with increasing concentrations of ICEC0942 or THZ1 for 48 hours, ICR-MCF7-942^R^ with the addition of ABCB1 inhibitors, verapamil or tariquidar (at indicated concentrations) (**B** ‑ **D**). Dose-response curves shown are from single representative experiments (error bars = SEM, *n* = 6). Dose-response curves from ICR-MCF7 and ICR-MCF7-942^R^ treated with ICEC0942 without the addition of ABCB1 inhibitors are shown in both B and C for clarity. ). Dose-response curves from ICR-MCF7 and ICR-MCF7-942^R^ treated with THZ1 without the addition of ABCB1 inhibitors are shown in both D and E for clarity Mean growth is shown relative to that for vehicle (DMSO)‑treated cells.

### Supplementary Figure S5. Overexpression of ABCB1 and ABCG2 results in resistance to CDK7 inhibitors.

The overexpression of ABCB1 in HEK293^ABCB1^ and ABCG2 in HEK293^ABCG2^ was confirmed by Western blot (A). HEK293, HEK293^ABCB1^ and HEK293^ABCG2^ were treated with increasing concentrations of THZ1 (B) and ICEC0942 (C) for 48 hours. Dose-response curves shown are from single representative experiments (error bars = SEM, *n* = 6). Mean growth is shown relative to that for vehicle (DMSO)‑treated cells.

### Supplementary Figure S6. Docking of ICEC0942 and THZ1 in the drug-binding pockets of human ABCB1 and ABCG2.

The transmembrane region of ABCB1 (grey helices) containing the lowest energy pose for each ligand, ICEC0942 (A) and THZ1 (B) is shown. The transmembrane regions of ABCG2 homodimer containing the lowest energy pose for each ligand, ICEC0942 (C) and THZ1 (B) is shown. Residues presented in gray sticks correspond to amino acids that are within 4.5 Å distance to the ligand. For ABCG2, the transmembrane helices of monomer 1 are shown in grey and monomer 2 in light blue. Figure was prepared using Pymol software. Docking scores of the nine lowest energy poses for binding of ICEC0942 and THZ1 to human ABCB1 and ABCG2 are shown in E.

**Supplementary Table S1. Primer sequences used in study.**
