# Supplementary material for: ABC-transporter upregulation mediates resistance to the CDK7 inhibitors THZ1 and ICEC0942
Source: Oncogene. 2019 Sep 17;39(3):651–63. doi: 10.1038/s41388-019-1008-y (PMC6962093; doi:10.1038/s41388-019-1008-y)
Supplement: Supplementary file 3 — Supplementary Figures. [file 41388_2019_1008_MOESM3_ESM.pdf]

Supplementary Figure S1.

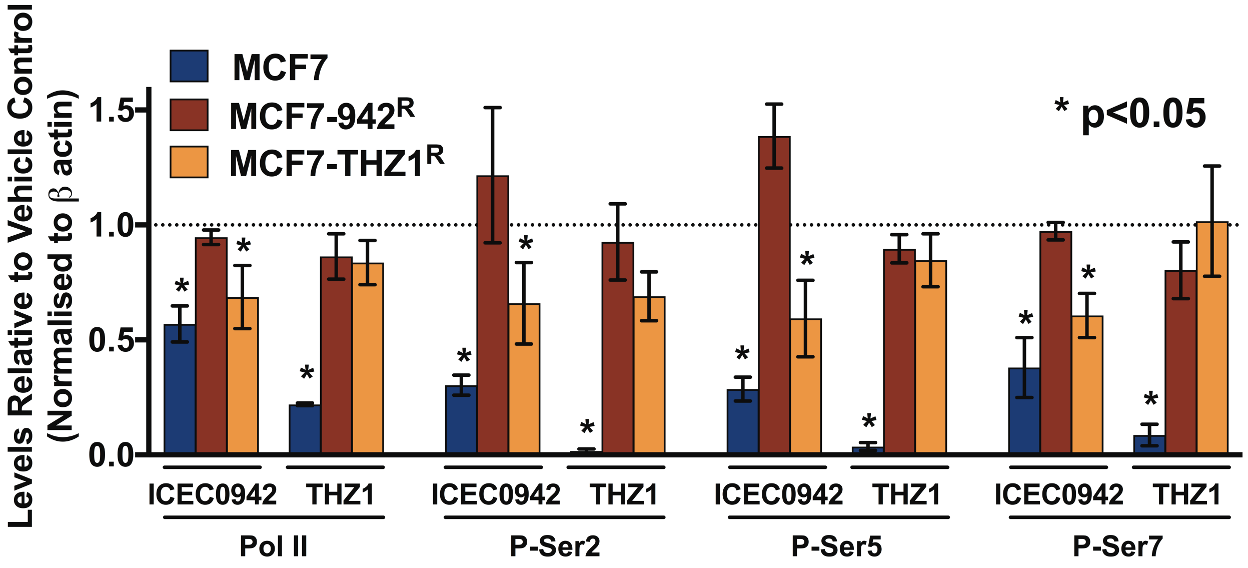

Supplementary Figure S2.

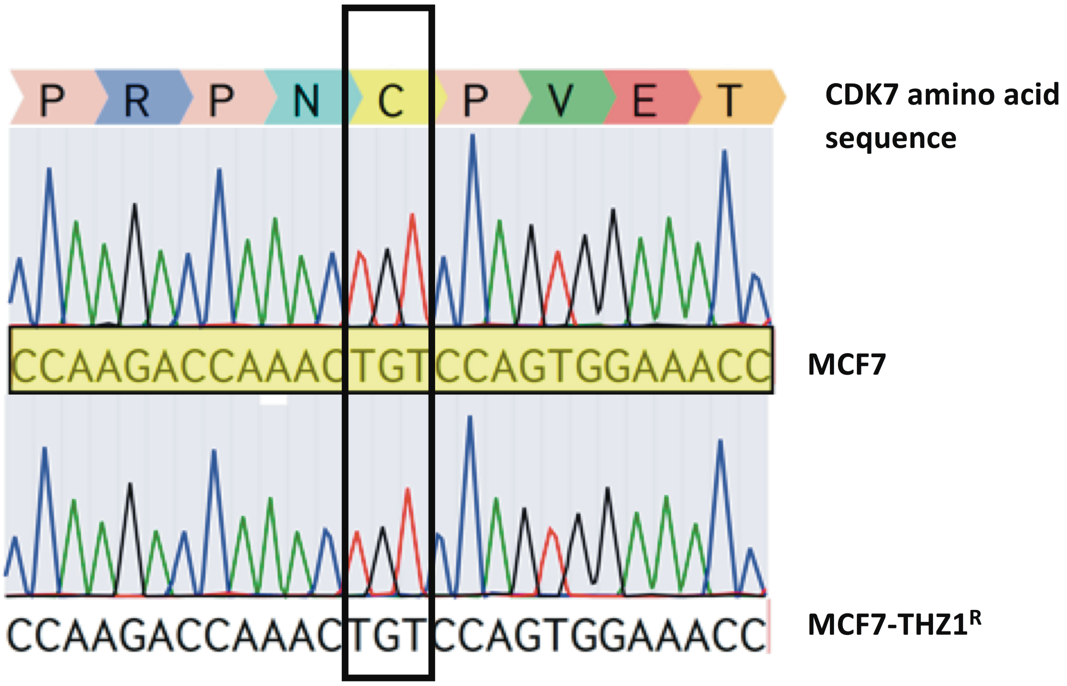

Supplementary Figure S3.

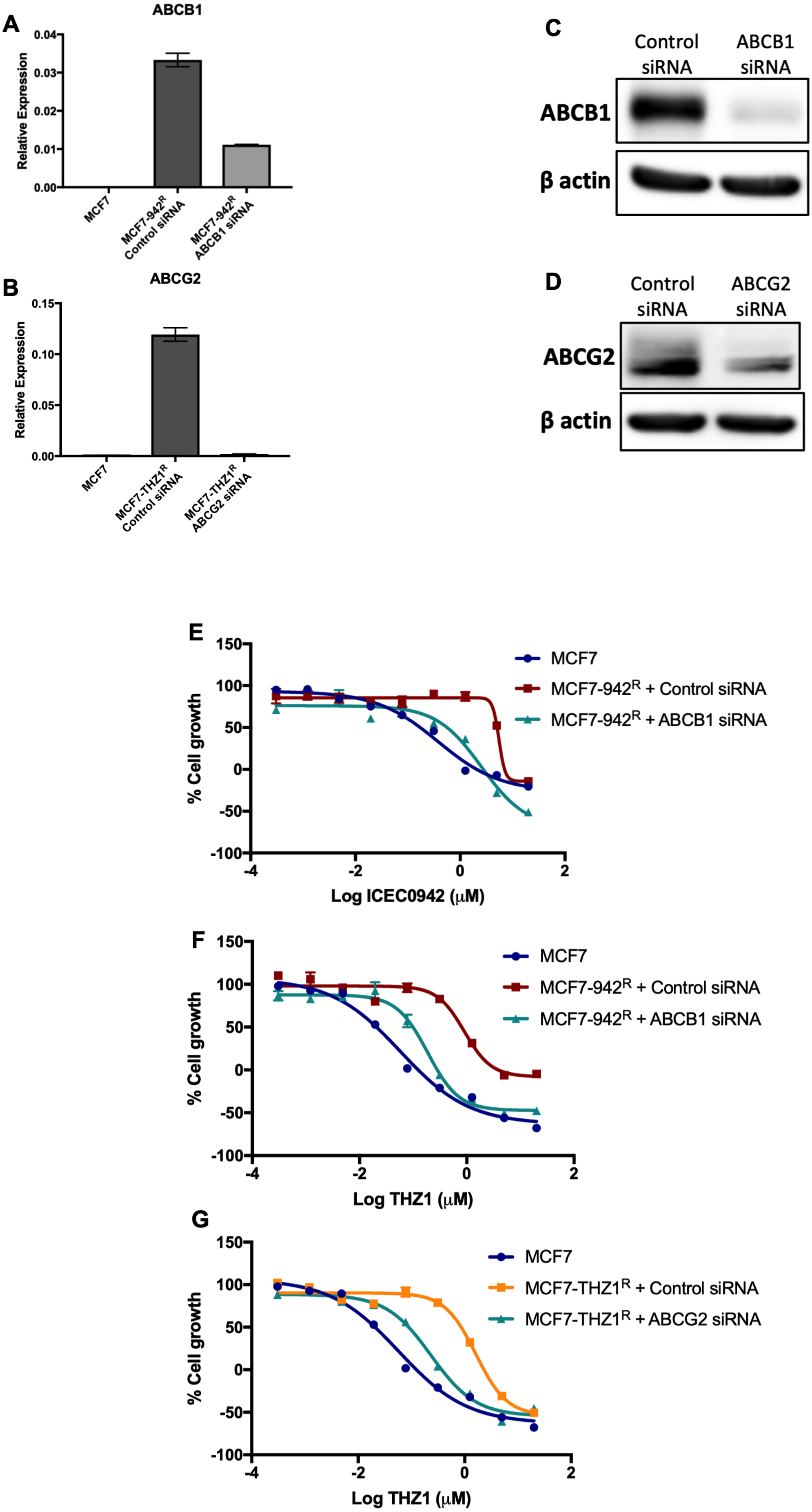

Supplementary Figure S4.

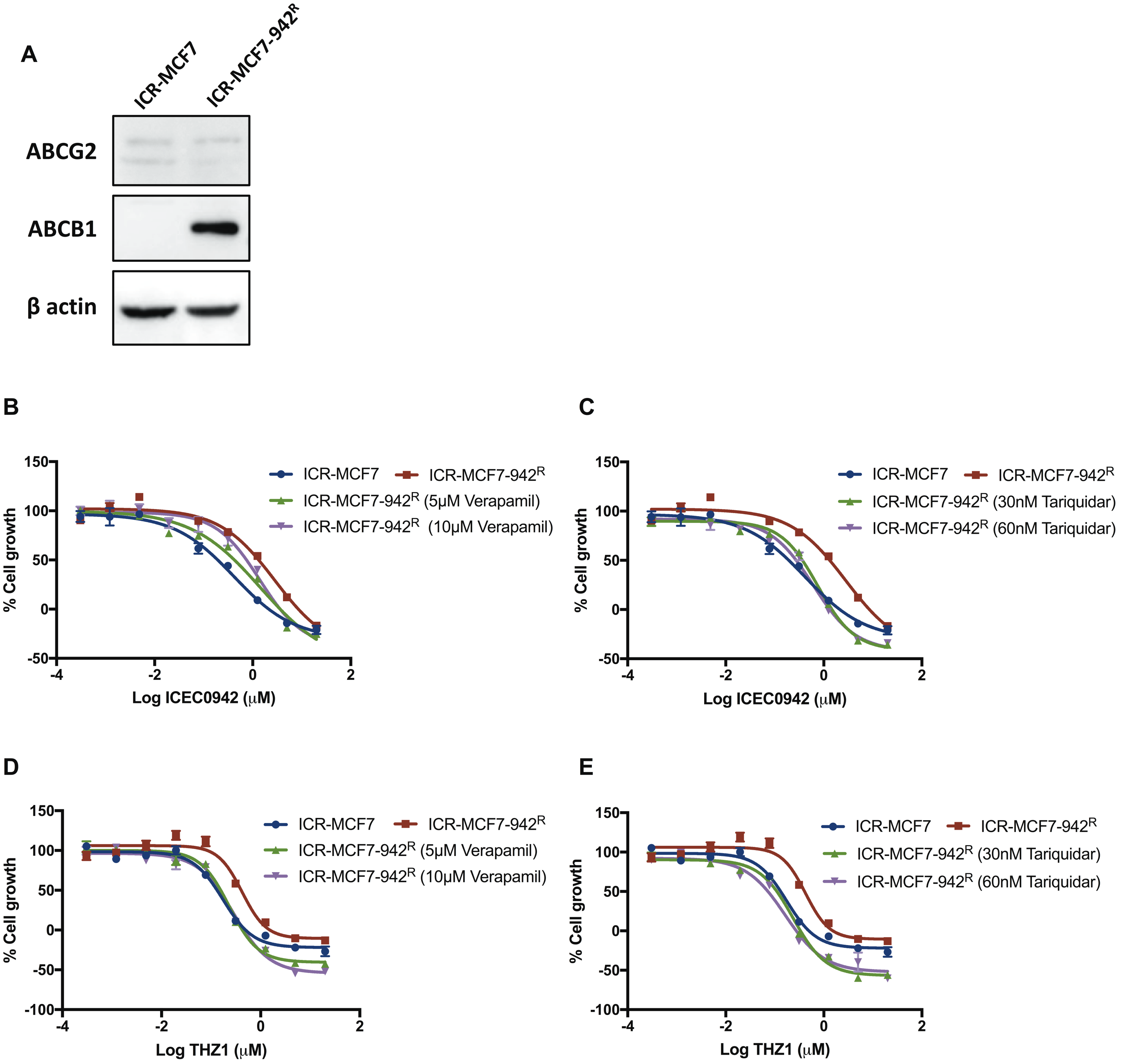

Supplementary Figure S5.

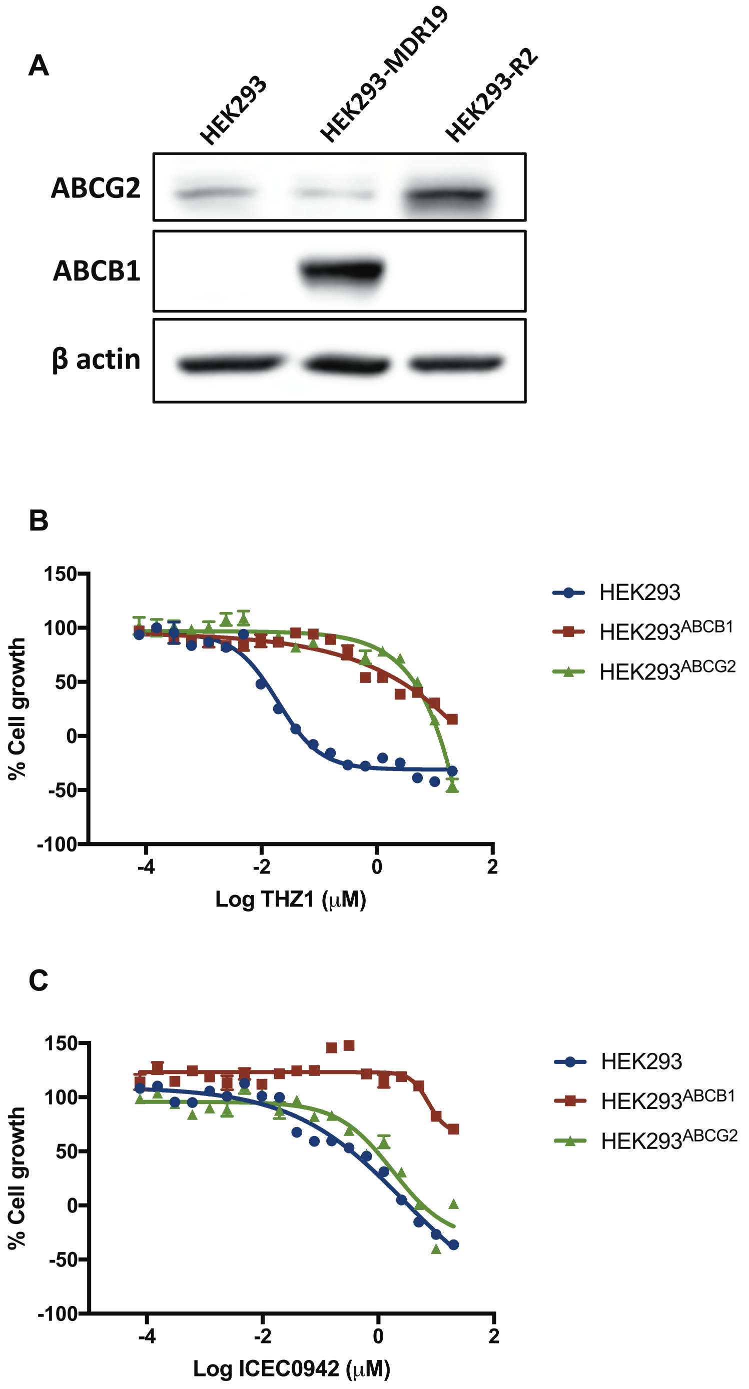

Supplementary Figure S6.

A

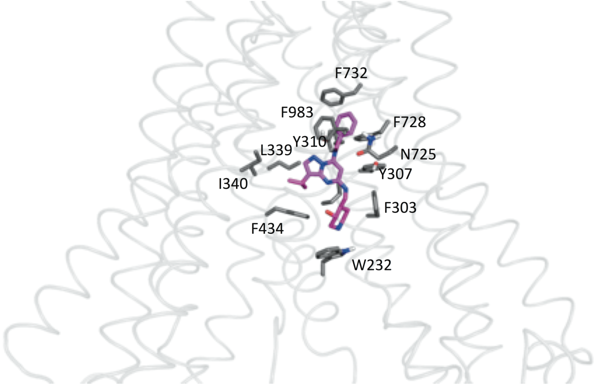

B

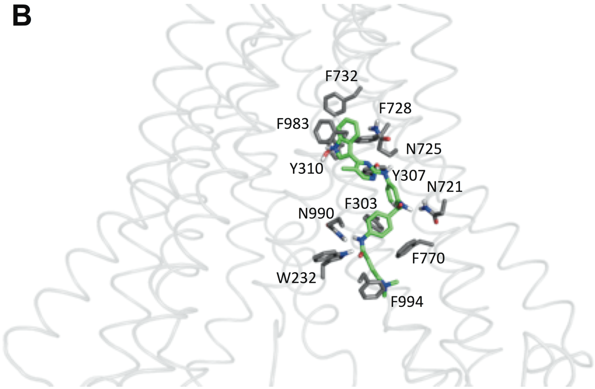

C

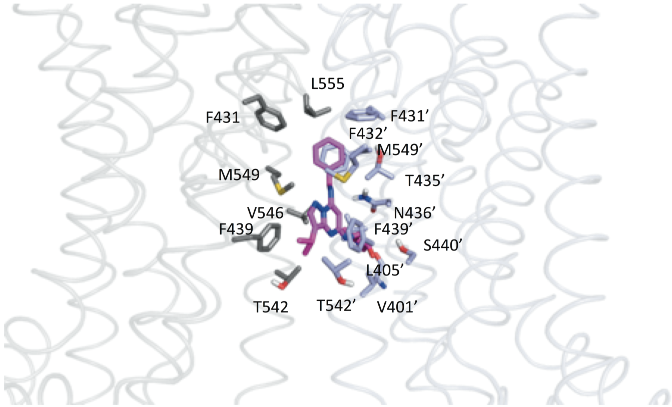

D

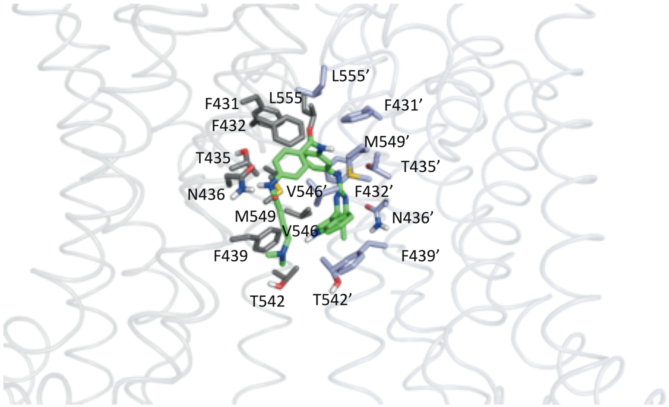

E

	Binding energy (kcal/mol)			
	ABCB1		ABCG2	
**Pose #**	**ICEC0942**	**THZ1**	**ICEC0942**	**THZ1**
**1**	-11.2	-14.3	-10.8	-13.7
**2**	-11.2	-14.2	-10.8	-13.5
**3**	-11.2	-14	-10.6	-13.2
**4**	-11.1	-13.8	-10.6	-13.1
**5**	-10.9	-13.8	-10.5	-13
**6**	-10.8	-13.4	-10.5	-12.9
**7**	-10.8	-13.3	-10.4	-12.9
**8**	-10.6	-13.3	-10.3	-12.9
**9**	-10.6	-13.3	-10.3	-12.8

Supplementary Table S1. Primer sequences used in study

**PCR**	
CDK7_C312_Forward	GCCCGGCCAGTCTTTTAATG
CDK7_C312_Reverse	ACGGCACAGTCTCTCTACTT
CDK7_UTR_Forward	AGTGGGTGTTGGAGGCTTTA
CDK7_UTR_Reverse	TGAACTTTTCTCACTTTTGCACT

**Sequencing**	
CDK7_C312_Forward	ACATTTCAGATACTCACCTACCT
CDK7_C312_Reverse	TGTTGTTCAGTCGCTAGCCA
CDK7_Forward_1	TCTCGGGCAAAGCGTTAT
CDK7_Forward_2	CCATCACACATCAAAGCCTACA
CDK7_Forward_3	ACACCAACTGAGGAACAGTG
CDK7_Reverse_1	CTTTGATGTGTGATGGTGTCAG
CDK7_Reverse_2	TCAGTTGGTGTGCCCAAAG
CDK7_Reverse_3	TGCCAAAGCTGGATTTGATTG

**qRT-PCR**	
ABCB1_F	TTGCTGCTTACATTCAGGTTTCA
ABCB1_R	AGCCTATCTCCTGTCGCATTA
ABCC1_F	CTCTATCTCTCCCGACATGACC
ABCC1_R	AGCAGACGATCCACAGCAAAA
ABCG2_F	ACGAACGGATTAACAGGGTCA
ABCG2_R	CTCCAGACACACCACGGAT
